# The Landscape of RNA-Protein Interactions in Plants: Approaches and Current Status

**DOI:** 10.3390/ijms22062845

**Published:** 2021-03-11

**Authors:** Vesper Burjoski, Anireddy S. N. Reddy

**Affiliations:** Department of Biology and Program in Cell and Molecular Biology, Colorado State University, Fort Collins, CO 80523, USA; Vesper.Craven@colostate.edu

**Keywords:** RNA binding proteins, RNA biology, plant science

## Abstract

RNAs transmit information from DNA to encode proteins that perform all cellular processes and regulate gene expression in multiple ways. From the time of synthesis to degradation, RNA molecules are associated with proteins called RNA-binding proteins (RBPs). The RBPs play diverse roles in many aspects of gene expression including pre-mRNA processing and post-transcriptional and translational regulation. In the last decade, the application of modern techniques to identify RNA–protein interactions with individual proteins, RNAs, and the whole transcriptome has led to the discovery of a hidden landscape of these interactions in plants. Global approaches such as RNA interactome capture (RIC) to identify proteins that bind protein-coding transcripts have led to the identification of close to 2000 putative RBPs in plants. Interestingly, many of these were found to be metabolic enzymes with no known canonical RNA-binding domains. Here, we review the methods used to analyze RNA–protein interactions in plants thus far and highlight the understanding of plant RNA–protein interactions these techniques have provided us. We also review some recent protein-centric, RNA-centric, and global approaches developed with non-plant systems and discuss their potential application to plants. We also provide an overview of results from classical studies of RNA–protein interaction in plants and discuss the significance of the increasingly evident ubiquity of RNA–protein interactions for the study of gene regulation and RNA biology in plants.

## 1. Introduction

DNA, the genetic blueprint of all organisms, controls all life processes through intermediate RNA molecules that dictate the types and levels of proteins made in cells. From the biogenesis to degradation of RNA molecules they are associated with many proteins. RNA–protein interactions are numerous, widespread, and play diverse biologically important roles in all organisms in many processes associated with gene regulation, including generation of coding and non-coding RNAs, transport, translation, and decay of RNAs, and control of diverse processes associated with development and disease. The proteins that interact with RNAs are collectively referred to as RNA-binding proteins (RBPs), a diverse class of proteins characterized by the presence of one or more RNA binding domains, usually alongside other catalytic or functional domains. Over 1800 candidate RBPs have been identified in plants, with over 800 enriched as RBPs in Arabidopsis [1]. Plant RBPs play diverse roles in growth, development, genome organization, stress response, immunity, mRNA processing, and post-transcriptional regulation [1,2,3,4,5,6,7]

RBPs rely on their RNA-binding domains to carry out their biological functions. Several of these classes of domains have been characterized, most notably the RNA-Recognition Motif (RRM), DEAD-box helicases, zinc finger domains, the K homology (KH) domain, the glycine-rich domains, the pentatricopeptide repeats (PPRs), and pumilio/fem-3 binding factors (PUFs) [1,7]. The RNA-binding domains allow RBPs to regulate many different processes: pre-mRNA splicing, pri-miRNA and pre-miRNA processing, polyadenylation, nuclear export, RNA stability, translation, RNA editing, etc. [2]. Moreover, many proteins identified as candidate RBPs lack classical RNA-binding domains, and there is even a high prevalence of metabolic enzymes identified as the RNA-interacting proteins, underscoring the complexity of RNA–protein interactions and the current gaps in understanding [8]. This is in accordance with results from mammalian mRNA-interactome studies, which revealed 23 distinct metabolic enzymes as RBPs [9]. Thus, it has been hypothesized that the moonlighting of metabolic enzymes as RBPs forms a regulatory link between cellular metabolism and RNA fate, known as the RNA-enzyme-metabolite (REM) hypothesis [9,10].

In this review, we begin by discussing the methods that are currently being used to directly probe RNA–protein interactions, highlighting those that have been used in plants and those that show potential to be used in plants. We discuss the advancements in knowledge contributed to plant biology by each method. We summarize our current understanding of RNA–protein interactions in plants as developed by the earlier use of classical genetics methods and touch on the current uses of RNA–protein interactions for biotechnological applications. Finally, we conclude with an outlook on the study of RNA–protein interactions in plants.

## 2. Overview of Methods to Detect RNA-Protein Interactions and Their Application to Plants

RNA-binding proteins have become a target of great interest in recent years, and many new methodologies have been developed to analyze the RNA–protein interactome. However, in plants, most research done in this field before ~10 years ago relied entirely on the use of indirect or in vitro methods to identify RNA and protein interaction, such as gel shift assay, mutant and knockout screening, nucleic acid-binding assay, and other classical genetics and cell biological techniques [2,11,12,13]. These techniques have contributed significantly to understanding the functions of RBPs in plant biology (see Section 3) but have since been superseded by the development of high throughput and global methods to analyze RNA and protein interactions. These new techniques were developed first in mammalian systems and a few have been used increasingly in plants. Below, we briefly describe these methods and their limitations, especially with respect to applying them in plants.

**Figure 1:**
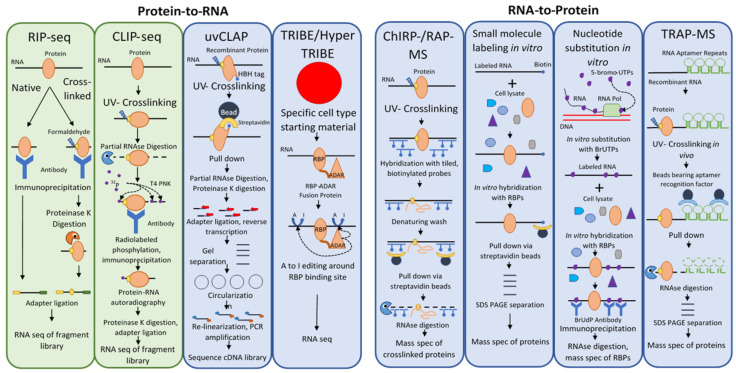
Methods to Detect RNA-Protein Interactions Using RNA or Protein Bait. Diagrammatic representation of the major steps in these notable methods. A green background denotes that the technique has been used in plants, whereas a blue background indicates that the technique has thus far only been used in mammals or other organisms. Crosslinking and immunoprecipitation (CLIP) derivatives are not detailed here but have been recently reviewed [14].

These techniques fall into three categories: (i) approaches that focus on identifying RNA targets of a candidate RBP, i.e., protein-to-RNA, (ii) approaches that focus on identifying the proteins interacting with an RNA of interest, i.e., RNA-to-protein, and (iii) global approaches (Figure 1). The vast majority of work that has been done in this field in plants has focused on the interacting partners of a single RNA or protein of interest (the bait), but recently the development of RNA-interactome capture (RIC) and its application to plants has allowed a global view of the plant RBPome.

### 2.1. Methods That Use a Protein Bait to Identify Its RNA Targets (Protein-to-RNA) and RBPs Identified Using These Methods in Plants

Among the first techniques developed to identify direct targets of RBPs in vivo was RNA immunoprecipitation or RIP [15,16] (Table 1). The basic idea of the RIP approach is simple and involves the use of an antibody against a protein of interest (Figure 1, RIP-seq). The lysate of cells expressing the protein of interest is incubated with antibody immobilized on beads, which are then washed and the proteins on the beads digested. The pool of RNA remaining is used to identify putative binding RNA targets. With the development of high throughput sequencing technologies, methodologies that used such sequencing platforms became known as RIP-seq [17].

RIP can also involve RNA–protein crosslinking, creating covalent bonds between the protein and its RNA ligands. Reversible crosslinking is accomplished using formaldehyde and reversed via heat treatment [18]. The drawbacks of this approach are that the specificity of the results depends on the strength of the antibody–protein interaction, and that formaldehyde treatment also catalyzes DNA-protein and protein–protein crosslinking, leading to the identification of indirect as well as direct targets of an RBP.

Crosslinking and immunoprecipitation (CLIP) builds on RIP by replacing formaldehyde crosslinking with UV-crosslinking to covalently link proteins with RNA molecules within several angstroms distance (i.e., bound by the protein) (Table 1). The RNA–protein complexes are selected after cell lysis using immunoprecipitation [19]. Partial digestion of the bound RNA allows a rough approximation of the binding site, followed by phosphorylation of the complexes with radio-isotope. The covalently bound RNA–protein complex is then rigorously washed, separated via SDS-PAGE, and transferred to a nitrocellulose membrane. The protein is then removed using proteinase K, linkers are ligated to the collected RNA fragments, and the fragment library is cloned after reverse transcription and then sequenced (Figure 1, CLIP-seq). There are many derivative techniques based on the basic CLIP-Seq principle. High-throughput sequencing of RNA isolated by crosslinking immunoprecipitation (HITS-CLIP) in place of traditional sequencing, which placed a limitation on the richness of data that could be generated by a CLIP-Seq experiment, allows more data to be extracted from CLIP fragment libraries (Table 1). This allowed the identification of over 1000-fold more unique binding sites compared to CLIP with traditional sequencing techniques, although this leaves one with the opposite problem—a plethora of data to sift through and discern signal from noise [20]. CLIP-based methods were further improved with the advent of CLIP experiments using photoactivatable ribonucleosides (PAR) to enhance the efficiency of crosslinking. PAR-CLIP incorporates 4-thiouridine into transcripts in vivo, which forms covalent bonds with interacting proteins under UV far more efficiently than random UV RNA–protein crosslinking; the approach improved RNA recovery 100- to 1000-fold [21].

Thus far, CLIP techniques were limited by the fact that reverse transcriptase often terminates prematurely when met with a residual amino acid covalently bound to a nucleotide at a crosslinking site causing such reads to be lost during standard CLIP library preparation. Individual-nucleotide resolution CLIP (iCLIP) was developed to compensate for this problem [22]. iCLIP captures truncated cDNAs using a cDNA self-circularization step in place of the previously used inefficient RNA ligation step in library preparation [22]. CLIP experiments also suffered from high experimental failure rates due to their technical complexity, and enhanced CLIP (eCLIP) was developed to address these issues. eCLIP decreases the amount of amplification necessary and uses random-mer barcode adapters ligated at the termination site of reverse transcriptase (the UV crosslinked nucleotide) to maintain analysis of RBP binding sites. Furthermore, the protocol omits the radiolabeling step and uses a size-matched control without immunoprecipitation to eliminate non-specific RNA interactions from the datasets [23].

The CLIP technique was also simplified by the development of simplified CLIP (sCLIP), which avoids radiolabeling by biotinylating the RNA for visualization, and uses polyadenylation and random-mer barcoding to uniquely identify RNAs and reduce the requirement for PCR amplification [24]. Another technique designed to avoid the use of radiolabeling, termed irCLIP for its use of an infrared dye, also used biotin labeling of the RNA—a biotinylated and infrared dye-conjugated 3’ adapter was ligated to the RNA, allowing visualization of RNA–protein complexes without autoradiography [25]. irCLIP allows the use of 250 times less starting material compared to iCLIP, and although comparisons were not performed with eCLIP or sCLIP, it seems likely that irCLIP lowers the starting material requirement most significantly.

With the advent of HITS-CLIP, many computational tools were developed in order to handle the large datasets produced by HITS-CLIP experiments. One of the most widely used of these is known as dCLIP, a program created to allow comparison of differential binding in different CLIP experiments [26]. dCLIP normalizes CLIP-seq data from different experiments using an application of a Bland-Altmann plot called an MA plot, then uses a Hidden Markov Model to detect shared or distinct binding sites across experiments. dCLIP has the advantage of being a universal computational tool for all types of CLIP-seq experiments; HITS-CLIP, PAR-CLIP, and iCLIP, and to allow comparison among them [26].

CLIP-Seq and its derivatives are powerful techniques but have significant limitations. Namely, CLIP (and its derivative methodologies) are all limited by their reliance on the antibody–antigen interaction; this limits the stringency of washing conditions to those that will not disrupt the antibody–antigen interaction [27]. Thus, the acquisition of a high-affinity antibody is critical for such experiments, and generally cannot be guaranteed. Even meaningful CLIP experiments contain significant noise in the form of proteins that were not eluted under the weak washing conditions, or in the form of proteins that co-immunoprecipitated [28]. Furthermore, CLIP relies on radiolabeling of bound RNA, a prohibitive procedure due to its cost, difficulty, and health hazards [29]. Finally, because of the low efficiency of CLIP techniques, they require large amounts of starting material, on the order of thousands of cells. This means that studies of RNA–protein complexes in specific cell types (which cannot be amassed in the thousands) are forced to use starting material of a mixed population of cell types, lowering the signal to noise ratio in their results [30]. Other techniques, discussed below, have been developed to avoid these limitations.

UV-crosslinking and affinity purification (uvCLAP) was developed as a radiolabeling- and immunoprecipitation-free alternative to CLIP methodologies [29] (Figure 1, uvCLAP). Instead of using an antibody–antigen interaction, uvCLAP relies on the tight interaction of the His6-biotinylation sequence-His6 (HBH) tag with beads that bind polyhistidine-tagged proteins, and then with the even more stringent interaction with streptavidin beads. The RNA is partially digested with RNAseI and the RNA ends are repaired with T4 polynucleotide kinase. Adapters are then ligated to the RNA fragments and reverse transcribed with barcoded primers. The cDNA products are then separated on a polyacrylamide gel, circularized to capture truncated cDNA products (as in iCLIP), linearized, and amplified with PCR.

The use of tandem affinity purification in this approach allows confidence that pulldown efficiency will be similar across conditions, experiments, and laboratories, in comparison with immunoprecipitation approaches in which every antibody–antigen interaction has a unique affinity [29]. uvCLAP also allows the quantification of nonspecific background noise, increasing its specificity. The drawback of this approach is the need for a genetic transformation with an HBH-fused construct prior to affinity purification. Although it is relatively unlikely when done carefully, such transformations could potentially alter RNA–protein interactions from their natural state. Moreover, this introduces extra steps for each RNA-binding protein studied; the significance of this drawback will depend entirely on the ease of genetic transformation in the model system being used.

The TRIBE (targets of RNA-binding proteins identified by editing) and HyperTRIBE approaches were developed in response to the severe limitations of CLIP-based techniques in identifying cell type-specific RNA–protein interactions [30]. TRIBE was developed first; it uses the RNA-editing enzyme ADAR’s (adenosine deaminase acting on RNA) to convert adenosines to guanines, leaving telltale signals in edited RNA (Figure 1, TRIBE/HyperTRIBE). In this approach, ADAR’s double-stranded RNA-binding motifs are replaced with the sequence of an RNA-binding protein of interest to create a fusion protein that targets ADAR’s RNA-editing activity to the RNA targets of the fused RBP. The RNA is sequenced, and detection of editing events indicates the binding of the fusion protein, and thus the RBP of interest.

The original TRIBE technique had the opposite problem as most CLIP experiments: it identified only about 25% of the target RNAs identified by CLIP techniques for the same RBP, and is thought to have had a false negative problem, rather than CLIP’s false positive problem. It was found that ADAR’s editing rate was low due to a sequence specificity for UAG and a double-stranded structure surrounding the edited adenosine [31].

To compensate for these weaknesses, hyperTRIBE was developed by introducing the E448Q mutation in ADAR, which lowers ADAR’s sequence and structure preferences and increases editing efficiency [31]. This mutation increased the number of detected editing events by over 20 times, while increasing the number of detected edited transcripts by 8 times. HyperTRIBE is able to identify about two-thirds of CLIP-identified target RNAs.

This approach has the advantages of avoiding the use of immunoprecipitation and radiolabeling, requiring only a small amount of starting material, and being simple. Like uvCLAP however, it also requires genetic transformation, and in comparison to both uvCLAP and CLIP techniques, has the drawback of providing no information as to the specific binding site on the RNA (as ADAR edits sites within up to 500 nucleotides of known CLIP sites). CLIP remains the method of choice if information about an RBP’s binding site on an RNA is desired, whereas HyperTRIBE is desirable if interested in RNA–protein complexes in specific cell types or if only small amounts of starting material are available [31].

Among the methods described above, the only protein-to-RNA techniques that have been used in plants to-date are RIP-seq and CLIP-seq. As discussed below, the application of these techniques to several RBPs has revealed their role in several processes.

#### 2.1.1. Regulation of RNA Processing

RIP-seq was used to demonstrate that the Arabidopsis Serine- Arginine-rich (SR) protein SR45 directly or indirectly associates with over 4000 RNAs in vivo, regulating constitutive and alternative splicing, post-splicing processing of 30% of ABA signaling genes, and over 300 intron-less RNAs [32] (Table 2). This indicates that SR45 exerts multimodal influence over mRNA processing, differentially regulating intron-containing and intron-less RNAs. The action of SR45 is defined by cis-elements in its RNA targets; four motifs were identified, two of which bear the hallmarks of exonic splicing regulators and two which showed peaks in the intronic regions of 5’ and 3’ splice sites. One of these motifs (M1; GAAGAA) was also found to be enriched in SR45’s intron-less targets [32]. Another study found 1812 RNAs associated with SR45, 81 of which were subject to alternative splicing mediated by the GGNGG motif in both activation and repression of splicing events [33]. These results further define SR45 as a splicing regulator whose activity cannot be easily defined as a positive or negative regulator, possibly explained by the fact that SR45 itself is alternatively spliced and its splice isoforms display differential expression. SR45 produces two splice isoforms, SR45.1 (long) and SR45.2 (short), the long isoform acting as a positive regulator in the salt stress response in Arabidopsis [34]. In rice, SR45 is stabilized through interactions with an immunophilin (OsFKBP20-1b), which plays an essential role in a positive regulation of transcription and splicing of stress response genes during abiotic stress [35]. THO2, a member of the Transcription-Export (THO/TREX) complex, was shown via RIP to participate in the generation of microRNAs; THO2 mutants showed both a decrease of miRNA accumulation and alterations in the splicing patterns of SR proteins, suggesting that the THO/TREX complex plays a role in alternative splicing [36].

RIP was used to show that the glycine-rich RBP AtGRP7 modulates alternative splicing in Arabidopsis [37]. A later study using both RIP-seq and iCLIP found 452 (RIP-seq) and 858 (iCLIP) RNA targets of AtGRP7 [38]. AtGRP7 alters the circadian regulation of its targets and seems to act in both alternative splicing and alternative polyadenylation (APA) [38] (Table 2).

Nuclear speckle RNA binding proteins (NSRs) have also been shown via RIP-seq to regulate mRNA processing, alternative splicing, and long noncoding RNA (lncRNA) prevalence [39] (Table 2). An NSR and an alternative splicing competitor (ASCO) lncRNA were shown to form a regulatory module of alternative splicing, in which the ASCO displaces an alternative splicing target from an NSR complex to modulate alternative splicing during development [39]. NSRs affected alternative splicing of hundreds of genes in Arabidopsis, and RIP-seq of an NSRa fusion protein showed that lncRNAs are also targets of NSRs, likely modulating their alternative polyadenylation or splicing as observed with the COOLAIR lncRNA to regulate cross-talk between auxin and immune response [40].

HITS-CLIP was used to identify genome-wide targets of HLP1, an hnRNP A/B protein that binds preferentially to A- and U-rich elements around cleavage and polyadenylation sites of transcripts involved in RNA metabolism and flowering to target APA [41] (Table 2). HLP1 suppresses Flowering Locus C (FLC) to release repression of flowering in Arabidopsis and control reproductive timing [41]. NSR knockout mutants showed modified APA and differential expression of the lncRNAs COOLAIR, produced from antisense transcripts generated from FLC, and function in the release of repression of flowering through suppression of FLC [40].

Using RIP-seq, the pentatricopeptide repeat protein PDM1 was shown to mediate cleavage of a transcript from polycistronic to monocistronic fragments in chloroplasts of Arabidopsis [42] (Table 2).

#### 2.1.2. Trafficking and Translocation

In rice, RIP-seq was used to show that RNA-binding protein-P (RBP-P) is an RNA-binding protein that plays a role in endosomal trafficking of glutelin and prolamine mRNAs, working to anchor the RBP-bound mRNAs to the endosome via the quaternary complex and transport it to the ER Subdomain for translation, coopting endosomal trafficking [43] (Table 2). RBP-L, an interacting partner of RBP-P, likely plays a coordinating role in subcellular trafficking of its mRNA targets, mediated by its 3’ UTR [44] (Table 2).

#### 2.1.3. Chaperoning

In Arabidopsis, a unique combination of RIP and microarray approaches (RIP-Chip) was used to demonstrate that the cold shock protein 1 (CSP1) acts as an RNA chaperone of polysomes to improve the translation of RNA targets at low temperatures [45] (Table 2).

#### 2.1.4. Gene Silencing

The RNA-directed DNA methylation effector KTF1 was identified via RIP as an RBP that binds Pol V scaffold transcripts to recruit argonaute 4 (AGO4) and its siRNAs for chromatin remodeling-mediated gene silencing [15] (Table 2). AGO4 and RNA polymerase V cooperate with 24 nt siRNAs in this process; siRNAs bound to AGO4 guide AGO4 to target loci through complementary base-pairing with nascent Pol V transcripts, where AGO4 recruits DNA modification factors such as DNA methyl-transferase DRM2 to methylate the chromatin and thus silence the affected genes [15,45] Based on RIP observations that the protein INVOLVED IN DE NOVO 2 (IDN2) is a lncRNA-binding protein that interacts with the SWItch/Sucrose Non-Fermentable (SWI/SNF) nucleosome remodeling complex, lncRNAs are thought to base-pair with siRNAs bound by AGO4 to position the SWI/SNF complex and thus target nucleosome remodeling, leading to decreased transcription by Pol II [46,47] (Table 2). RIP was also shown to be usable in Arabidopsis for the detection of lncRNAs generated by specialized polymerases [48].

#### 2.1.5. Viral RNA Suppression

A modified RIP-seq assay was developed for the detection of RNAs of heterologous origin in plants and applied to transiently expressed nuclear epitope-containing proteins in *Nicotiana benthamiana*, but to-date this method has not been used for its intended purpose of detecting viral RNAs in plant cells [49].

#### 2.1.6. Other RBPs

The plastid UMP kinase (PUMPKIN) has been shown via RIP-seq to associate with several RNAs in vivo, altering their metabolism thereby [50] (Table 2). This suggests that while PUMPKIN is primarily a metabolic enzyme, it may have a moonlighting function as an RBP, potentially for the purpose of coupling RNA and pyrimidine metabolism [50].

#### 2.1.7. Perspective on the Application of Protein-to-RNA Methods in Plants

Despite the breadth of techniques available for use in elucidating RNA–protein interactions in vivo, CLIP and its derivatives remain the most tenable non-global approach for use in plants. RIP has also been used extensively and is suitable for certain experimental purposes. There still remain several techniques used in other organisms to probe interactions between a protein of interest and RNAs that have yet to be successfully adapted, or even tried, in plants. These are opportunities for advancement in plant RNA biology, but if adapted into plants should be modified to include the best features and optimizations of the already-proven RIP and CLIP approaches.

**Figure 2:**
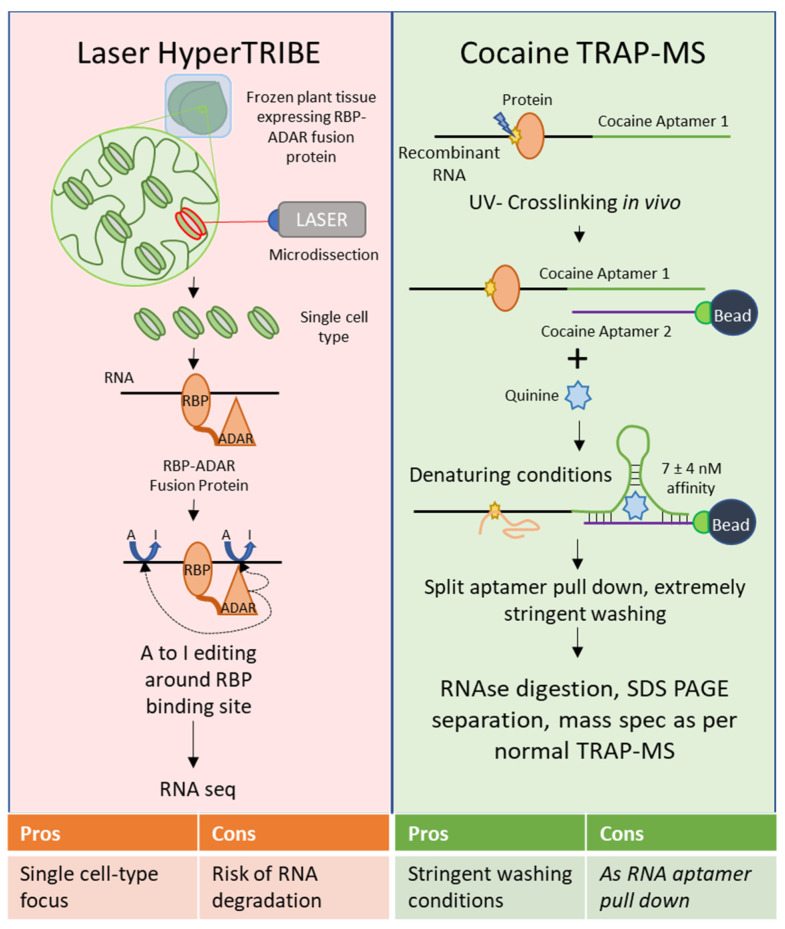
Proposed Modifications to Existing Techniques for Future Advancement. Laser HyperTRIBE—This is not a true modification to the HyperTRIBE approach itself, but rather a pairing of that approach with laser microdissection to enable the strengths of HyperTRIBE (capacity to accommodate small amounts of starting material) to be applied to plants. In this approach, intact plant tissue would be fixed prior to flash freezing, and then the cell types of interest selected via laser microdissection used for HyperTRIBE. Cocaine TRAP-MS—This modification suggests that rather than RNA aptamers that rely on protein binding capabilities, nucleotide-nucleotide aptamers should be prioritized. The split cocaine aptamer consists of two RNA fragments that interact at very high affinity in the presence of cocaine, or preferably quinine thanks to its increased affinity and ease of access. The high affinity of this interaction would permit extremely stringent washing conditions, which are critical in minimizing false positives.

Several of these techniques show particular promise; TRIBE, and particularly HyperTRIBE, have not been used in plants as yet, but if viable would overcome the signal to noise ratio problems inherent in CLIP. HyperTRIBE outperforms CLIP when using a small amount of starting material, such as a few cells of homogenous origin. Unfortunately, techniques used to select cells of a single type from a heterogeneous sample in mammalian systems, such as flow cytometry, are not tenable in plants without significantly altering the cell state (i.e., generating protoplasts by degrading the cell wall) [51]. Laser microdissection of plant tissues seems the most viable route for selecting cells of a particular type in plants, and HyperTRIBE would allow the use of smaller amounts of starting material than were previously used for RNA-Seq after laser microdissection [52]. Focus on single cell-types is a necessary next step for plant biology to throw off the albatross of whole-plant and tissue heterogeneity, and HyperTRIBE combined with laser microdissection would represent progress toward that goal in the field of RBPomics (Figure 2). However, laser microdissection requires a somewhat more extended time between sample harvesting and freezing due to the fixation step, which could result in increased RNA degradation after harvesting. Even so, transcriptional profiling has been performed successfully using cells harvested via this technique [52].

### 2.2. Methods That Use an RNA Bait to Identify Binding Proteins (RNA-to-Protein)

RNA antisense purification mass spectrometry (RAP-MS) is a technique used to purify long noncoding RNAs and their interacting proteins with complementary, tiled, biotinylated DNA probes bound to magnetic streptavidin beads [53] (Figure 1, ChIRP-/RAP-MS). RAP-MS starts with UV crosslinking of RNA to interacting proteins in vivo. The crosslinked RNA–protein complexes are then extracted under denaturing conditions to disrupt non-covalent interactions, and the complexes are hybridized with ~120 nt biotinylated DNA probes bound to magnetic beads. After washing, the RNA is digested, and the protein pool is analyzed using mass spectrometry (MS). This method also uses stable isotope labeling by amino acids in culture (SILAC) to label proteins, allowing quantitative comparisons to be made with mass spectrometry [54].

Comprehensive identification of RNA-binding proteins by mass spectrometry (ChIRP-MS) is a related technique predating RAP-MS by several years [55]. It also uses tiled biotinylated DNA probes bound to magnetic streptavidin beads and RNA–protein crosslinking, although the probes used were only 20 nt in length and formaldehyde crosslinking was chosen instead of UV crosslinking. The use of formaldehyde crosslinking has the advantage of being reversible, and ChIRP-MS studies are able to reverse crosslinking while keeping both protein and RNA components intact and allowing further analyses on both [56]. However, formaldehyde crosslinking also catalyzes the crosslinking of protein–protein and protein-DNA interactions.

The technique known as PIP-Seq has been used successfully to elucidate important RNA–protein interactions governing the differentiation of root hair cells [57]. PIP-seq identifies RNA–protein interactions with precise RNA binding sites when paired with a technique capable of identifying individual interacting RBPs. PIP-seq uses formaldehyde crosslinking to covalently bond RNA to interacting proteins, followed by high-throughput sequencing. The sample is split into a matrix of four: one sample with RBPs intact treated with single-stranded RNA nuclease (ssRNAse), one without RBPs treated with ssRNAse, one with RBPs treated with double-stranded RNA nuclease (dsRNAse), and one without RBPs treated with dsRNAse. The use of ss- and dsRNAse in the presence and absence of binding RBPs allows both RNA structure and RBP protection (and thus binding) to be predicted [57].

Recently, a CRISPR-based system called CRUIS (CRISPR-based RNA-United Interaction System) was developed in mammals [58]. CRUIS uses transient expression to couple the RNA-tracking capabilities of dCas13a with a fused proximity protein, Pafa, which labels surrounding RNA-binding proteins. These labeled proteins can then be identified via mass spectrometry. CRUIS was shown to be roughly as efficient as CLIP and identified novel protein targets [58]. The advantage of this technique is that it captures truly in vivo interactions without the potential for spurious interactions to form during lysis and wash steps, but it remains to be seen whether it has a false positive problem. The Pafa proximity labeling protein lacks the specificity of UV crosslinking for angstrom-level RNA–protein interactions, potentially leading to the labeling of indirectly interacting proteins.

There are a number of RNA to protein methods that are useful for in vitro studies but are not applicable to in vivo work. Among these is the labeling of RNA with small molecules [59]. In this RNA to protein approach, small molecules are covalently bonded to an RNA of interest in vitro, then incubated with cell lysate and pulled down using an immobilized receptor for the small molecule ligand (Figure 1, small molecule labeling in vitro). Common forms of this technique include biotin labeling, desthiobiotin labeling, and digoxigenin labeling. Unfortunately, because of the chemical reactions necessary to label an RNA of interest, small molecule RNA labeling is usually not appropriate for in vivo studies.

Another exclusively in vitro approach is nucleotide substitution in RNA [59]. Here, RNA is transcribed in vitro in the presence of a heavy metal-modified dNTP, incorporating the modified nucleotide into the transcript. Immunoprecipitation can then be carried out using an antibody against the modified nucleotide (Figure 1, nucleotide substitution in vitro). The drawback of this approach is that the charge of the heavy metal-modified nucleotide can strongly affect the charge distribution, structure, and protein binding of the RNA of interest.

Whereas the uvCLAP approach uses modifications to the protein primary structure, RNA aptamer pulldown (also known as tandem repeat affinity purification mass spectrometry, or TRAP-MS) uses modifications to the RNA primary and secondary structures, followed by tandem affinity purification [59]. RNA aptamers are short oligonucleotide sequences that reliably assume a secondary structure under physiological conditions, which tightly interacts with a target molecule—the ligand. The affinities of these interactions can be equivalent to or greater than those of antibody–antigen interactions [60,61,62,63]. An RNA aptamer is introduced into an RNA of interest either in vitro or in vivo, the lysate is passed over a column containing immobilized ligand, washed, and ribonucleoprotein complexes are eluted. Interacting proteins are identified via mass spectrometry. This, like RAP-MS/ChIRP-MS, is one of the few in vivo methods to identify ribonucleoprotein complexes in the RNA-to-protein direction.

There are many well-studied RNA aptamers used for such studies; some of the most commonly used are the PP7, S1, D8, tobramycin, streptomycin, Csy4 (H29A), Mango, and MS2 aptamers [59,60,61,62]. Only the MS2 aptamer will be discussed in detail here. This aptamer exploits the tight, highly specific interaction between the coat protein (MCP) of the bacteriophage MS2 and a 19nt RNA hairpin structure from the bacteriophage’s genome, which the virus presents on the surface of its genome to assemble its coat protein [60]. Repeats of the MS2 hairpin structure are inserted at the 3’ end of an RNA of interest, while a fusion protein of MCP and maltose-binding protein (MBP) is immobilized on amylose beads. After pulldown, the protein-RNA-MCP-MBP complex is eluted using excess maltose, which MBP binds preferentially (Figure 1, TRAP-MS). RNA aptamer pulldown has the disadvantage of requiring genetic transformation, which may alter the structure of the RNA of interest and thus distort the pool of RNA binding proteins associated with it. Furthermore, the presence of the RNA aptamer may risk aggregation.

Two other RNA-to-protein techniques were developed in the last year in non-plant systems. One of these methods targets engineered peroxidase (APEX) with MS2 or Cas13 to a specific RNA. APEX targeting uses either the MS2-MCP interaction or an engineered CRISPR-Cas13 interaction to target the biotinylation activity of APEX2 to proteins proximal to target RNAs in vivo [64]. After rapid, one-minute biotin labeling, cells are lysed and pulled down using streptavidin beads. Isolated proteins are identified using liquid chromatography-mass spectrometry (LC-MS). This method was based on the RNA proximity biotinylation (RNA-BioID) and APEX RNA immunoprecipitation (APEX-RIP) approaches. RNA-BioID uses MCP to target a biotin ligase (BirA*) to an MS2-tagged RNA of interest [65]. APEX-RIP uses the promiscuous engineered peroxidase APEX2 expressed by live cells to target cellular components of interest and biotinylate proximal proteins during a short pulse of treatment with hydrogen peroxide and biotin-phenol [66]. Following biotinylation, labeled proteins are crosslinked to proximal RNAs using formaldehyde and pulled down using streptavidin beads, along with co-eluting RNAs. APEX targeting improves on BioID by decreasing the amount of time necessary for biotin labeling [66]. Although it is claimed [66] that APEX2 does not label distal proteins due to the short half-life of the biotin-phenoxyl radical it generates, it is unknown whether APEX2 may label proteins interacting indirectly with the target RNA. Compared to crosslinking, which establishes a hard limit on the distance of RNA–protein interaction, this may raise a concern of false positives when using APEX targeting.

The second method is called CRISPR-assisted RNA–protein interaction detection (CARPID). This method was also inspired by APEX-based approaches but uses the engineered biotin ligase BASU instead of APEX2 [67]. Using a nuclease-activity-free RNA targeting dCasRx to tether BASU to RNAs of interest, CARPID labels interacting proteins via biotinylation, followed by pull-down with streptavidin beads [67]. This method was able to identify RBPs interacting with lncRNAs but requires a longer labeling period as compared to APEX targeting.

Perspective on the Use of RNA-to-Protein in Plants

There is much room for improvement in the RNA-to-protein direction, particularly considering that none of these techniques have been used in plants to-date. ChIRP-MS in particular would be an attractive technique to attempt in plants for the following reasons: it avoids the need for antibody generation used in RIP and CLIP, it does not use radiolabeling, it permits denaturing conditions and stringent washes, and it does not require genetic transformation. However, as previously described it cannot provide any information regarding the binding site of an RBP.

RNA aptamer-mediated pull-down techniques could also be an area for advancement. These approaches do require genetic transformation and could potentially result in altered RNA secondary structure (depending on the aptamer used), but their potential to exceed the antibody–antigen affinity limitations and avoid the antibody generation variabilities of CLIP makes them attractive nevertheless. However, because most of the annotated RNA aptamers in use rely on the binding capabilities of partner proteins (such as the MS2 stem loop’s binding by the MS2 viral coat protein MCP), their use limits the stringency of washing conditions; denaturing conditions cannot be used during incubation and washing to prevent the formation of post-lysis ribonucleoprotein complexes without denaturing the aptamer’s binding partner and thereby compromising pull down. Of those that do not rely on protein partners, few match the affinity granted by the polyA-oligo(dT) interactions used in other techniques, such as RNA-interactome capture.

It might be advantageous to develop nucleotide-nucleotide RNA aptamers to increase the binding affinity, such as by applying a split RNA aptamer. Split aptamer approaches involve separating out an existing aptamer, such as an RNA that forms a tight stem-loop secondary structure, into two fragments that tightly interact in the presence of a ligand; thus, one fragment of the aptamer is appended to a transcript of interest, and the second is immobilized on a nonreactive bead. For example, the cocaine aptamer has successfully been split and used as a biosensor [68]. Although its target as a biosensor is cocaine, the split aptamer actually shows 30- to 50-fold greater affinity for quinine over cocaine, binding at an affinity of 7 ± 4 nM [68,69]. Use of the cocaine aptamer in the presence of quinine during pull-down could potentially tolerate extremely stringent washing conditions. For a summary of these suggested techniques, see Figure 2. Proximity biotinylation-based methods, such as APEX targeting and CARPID could theoretically be used in plants and would be of interest due to their status as RNA-to-protein methods with some modifications.

### 2.3. Global RNA-Protein Interactome

**Figure 3:**
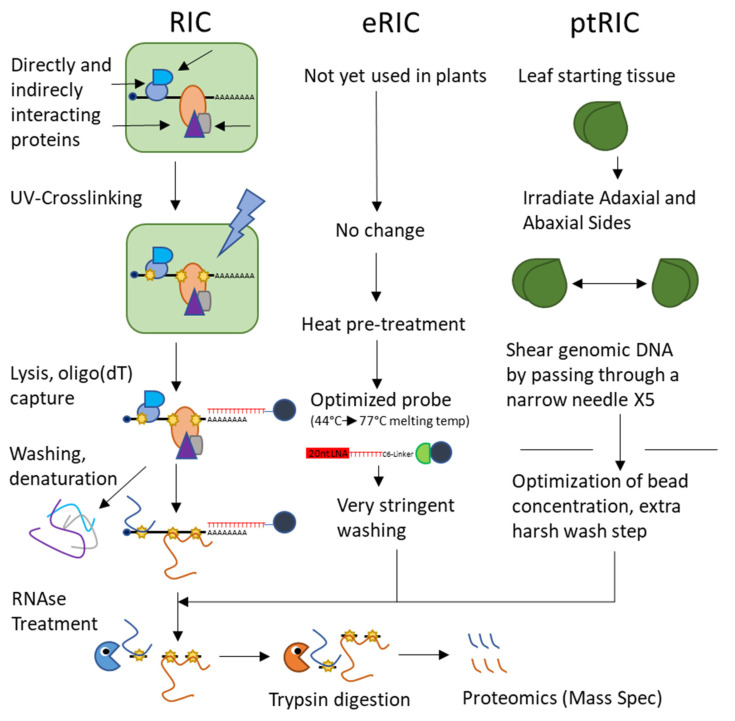
RNA Interactome Approaches for RBPome Investigation. The basic RNA interactome approach involves UV-crosslinking (either traditional or using photoactivatable nucleoside 4SU) to covalently bond RNA to interacting proteins, cell lysis, pulldown of polyA RNA using oligo(dT) conjugated beads, followed by stringent washing, denaturation of proteins to eliminate non-crosslinked interactions, and then RNAse treatment to remove RNA, trypsin digestion, and mass spectrometry proteomics to identify the crosslinked protein cohort. Enhanced RIC (eRIC, not yet used in plants) uses a heat pre-treatment to dissociate non-crosslinked proteins and an optimized DNA probe conjugated to beads instead of standard oligo(dT) beads. The optimized probe permits very stringent washing conditions, which eliminates background. ptRIC was developed specifically for plants and optimizes irradiation conditions by repeatedly irradiating both sides of the leaves, removing genomic DNA by shearing, and optimizing bead concentration. There is no apparent barrier to combining the eRIC and ptRIC approaches to generate a super-optimized RIC protocol for plants.

Until very recently, there was only one currently available global approach to capturing the plant RBPome, called RNA-interactome capture (RIC). RIC uses techniques common to directed RNA–protein interaction studies, beginning with the UV-crosslinking of interacting proteins to their partner RNAs as in CLIP and PAR-CLIP. The cell lysate is then passed over oligo(dT)-magnetic beads under denaturing conditions to pull down polyA RNAs and the denatured proteins covalently bonded to them. After stringent washes to elute any non-covalently interacting proteins, the RNA is enzymatically digested and the protein sample is subjected for proteomics via mass spectrometry [70] (Figure 3). This technique is powerful but limited by its restriction to polyA RNA.

The RIC technique was adapted into plants several years ago by a trio of studies using cell suspension cultures, seedling leaves, leaf mesophyll protoplasts, and etiolated whole seedlings [8,71,72]. These studies identified between 300 and 1200 RBPs, all showing enrichment of proteins containing canonical RNA-binding domains. They also all identified a significant proportion of proteins lacking a canonical RNA-binding domain and playing no known role in RNA biology, underscoring how poorly described RNA–protein interactions are in plants. Finally, all three studies found significant proportions of enzymes involved in intermediate metabolism making up the RBPome, suggesting that the RNA-enzyme-metabolite hypothesis may be a valid consideration in plants as well as mammals.

These studies provide us with a perspective on heterogenous plant samples grown under normal conditions and provide a baseline against which future studies may compare the results of experiments using the array of sample types described. Since their publication, the RIC method has been applied to Arabidopsis cell cultures grown under drought stress (using PEG to simulate drought conditions in culture) to identify 150 RBPs responsive to drought stress [73]. Similarly, RIC was used to probe modifications of the spliceosome and its RBPs in response to drought, identifying 44 spliceosomal proteins and 32 proteins associated with stress granules [74]. Like the previous studies, this work identified many metabolic enzymes interacting with RNA, comprising proteins involved in carbohydrate metabolism and the glycolytic and citric acid pathways. Recently, several optimizations of the RIC protocol—deemed enhanced RNA interactome capture or eRIC—were described, but these modifications have yet to be applied to plants [75] (Figure 3). Separately, RIC has been optimized for leaf tissue (termed plant RNA interactome capture or ptRIC) by adjusting UV conditions, irradiating both adaxial and abaxial surfaces of leaves, increasing the stringency of washing conditions, and shearing genomic DNA by passing the RNA-loaded beads through a narrow needle [76] (Figure 3). It remains now for RIC or its derivatives to be used to view the changes of the RBPome in response to biotic and abiotic stresses beyond drought.

Very recently, a new method for the identification of RNA–protein interactions has been adapted from bacterial and mammalian systems, known as orthogonal Organic Phase Separation, or OOPS. This method uses UV-crosslinking, similar to other techniques, and acidic guanidiniumthiocyanate-phenol-chloroform (AGPC) phase separation to collect RBPs at the interface between the aqueous and organic phases [77]. OOPs has the advantage of being simpler than many other techniques and of not requiring mRNA pulldown, thus capturing RBP interactions with all types of RNA rather than solely coding RNAs. OOPS was applied in Arabidopsis to identify 468 RBPs, 232 of which were enzymatic putative RBPs [78].

## 3. RNA-Protein Interactions in Plants as Unearthed by Classical Methods

**Figure 4:**
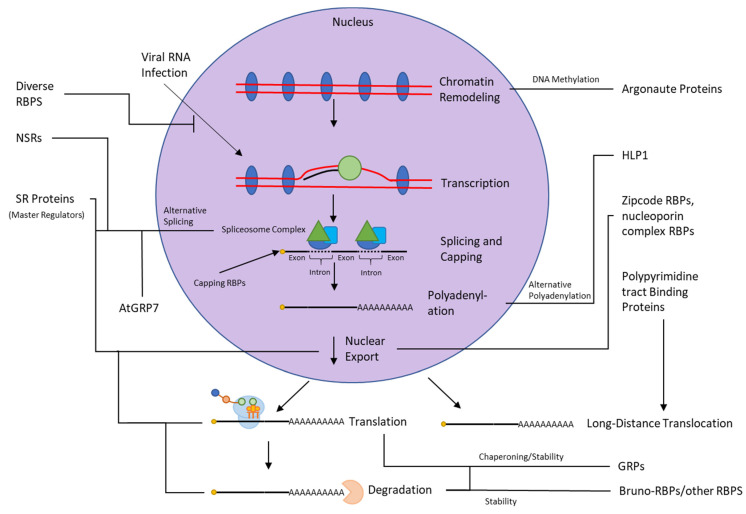
Summary of Known RNA-Protein Interactions in Plants. RNA-Binding Proteins play significant roles in carrying out and regulating every step and stage of the existence of RNAs. This diagram illustrates currently known RBPs and their RNA-binding roles, highlighting their ubiquity. Most of these RBP classes are capable of both positive and negative regulation, and as such lines indicating their activity have been left without arrows or bars.

The study of RNA–protein interactions predates the use of the methods discussed above. Before the advent of techniques capable of directly establishing RNA–protein interaction in vivo, classical genetics and cell biological methods were used to compile evidence supporting the interaction of RNA and protein. To provide a comprehensive understanding of plant RBPs role in diverse cellular processes, we reviewed the literature using classical methods to probe RNA–protein interactions in plants (Supplementary Table S1). The totality of our current understanding of RNA–protein interactions in plants, derived from both modern and classical methods, is summarized in Figure 4. 

### 3.1. Regulation of RNA Processing

In all eukaryotes, RNAs are processed with a 5’ methylguanosine cap shortly after RNA polymerase synthesizes the first 25–30 nucleotides; capping is accomplished by the catalytic activities of RBPs, and the cap subsequently increases stability and participates in pre-mRNA splicing via cap binding proteins [79]. Next, exons are spliced together by the spliceosome complex, which is composed of a small nuclear ribonucleoprotein (snRNP) complex containing multiple RBPs; the U2 snRNP auxiliary factors U2AF35 and U2AF65 subunits bind to the intron/exon borders to mediate cleavage at both splice sites and then ligation [80]. Alternative splicing of exons can regulate transcript abundance by generating premature termination codons or unstable mRNA isoforms targeted for nonsense-mediated degradation, and novel protein isoforms may be produced in response to stress to alter protein localization or function [81,82].

Fully transcribed and spliced mRNAs are polyadenylated at the end of the 3’ UTR (although alternative polyadenylation sites with significance in transcript stability, expression, and regulation have been observed), by the polyadenylation complex which is composed of a large number of proteins, some of which are RBPs [83,84]. Finally, the ribosome itself is a ribonucleoprotein complex containing at least 80 ribosomal proteins, many of which are RBPs [85].

Regulation of RNA processing is mediated by RBPs at multiple levels. As discussed in Section 2, SRs and NSRs participate in the regulation of alternative splicing and polyadenylation. In *Medicago truncatula*, NSRs are known to partner with the lncRNA early nodulin 40 (ENOD40) involved with root nodulation [86]. NSRs are quite ancient, predating the rise of true vascular plants, and have shown a reductive evolutionary trend in eudicots compared to other angiosperms, sometimes limited to as few as one or two genes [87]. This phylogenetic analysis showed that there are three motifs conserved across NSRs in plants: a nuclear localization signal, a motif of unknown function, and the C-terminal RRM. It has been suggested that alternative splicing of NSRs may help compensate for their reduction in eudicots [87].

Splicing of plant resistance genes is controlled by modifier of snc1,12 (MOS12), a cyclin L homolog, and the MOS4-associated complex (MAC), localized to the nucleus [88]. The MAC is a nuclear complex that also comprises the Arabidopsis homolog of cell cycle serine/threonine-protein kinase (AtCDC5) transcription factor and protein pleiotropic regulatory locus (1PRL1), a beta-transducin repeat (WD-40) protein [89]. Immuno-affinity purification of the MAC followed by proteomics identified 24 MAC-interacting proteins, most of which are predicted to participate in RNA processing, including U2 and U5 subunits and several other RBPs [90].

The splicing modulator AtGRP7, shown by RIP to modulate alternative splicing (Section 2), is regulated by the circadian clock and downregulates FLC, as indicated by AtGRP7 knockout and overexpression lines, to control flowering time [37,91,92]. AtGRP7 also controls alternative splicing of the flowering locus M (FLM) floral repressor to act in the thermosensory control of flowering time [93]. A paralog of AtGRP7, AtGRP8, coordinates with AtGRP7 in the alternative splicing of FLM, potentially playing a stronger role in that process that AtGRP7 [93]. The circadian-clock functions of AtGRP7 are controlled by its RNA binding domain as determined by transcriptional profiling of lines expressing AtGRP7 with a point mutation in the RNA binding domain [37,91,94]. AtGRP7 is a target of a bacterial type III-secreted effector, HopU1, to suppress plant immunity via ADP-ribosylation of AtGRP7’s RNA binding site and thus interfering with its ability to bind RNA [95].

In maize, the nuclear-localized RRM protein defective kernels 42 (Dek42) impacts alternative splicing through its interaction with spliceosome components, including splicing factor 3a subunit 1 (SF3a1) and U1 small nuclear ribonucleoprotein 70 kDa (U1-70k) [96].

Alternative polyadenylation (APA) forms another mechanism of regulation by RBPs, which produces mRNAs with distinct 3’ ends by modifying cleavage and polyadenylation sites in the 3’ UTR [97]. Over 70% of mammalian transcripts have APA isoforms, and over 50% of plant transcripts [97,98]. The RBP known as flowering time control protein (FPA) is involved in FLC regulation repression to enable flowering, involved in 3’ end processing and alternative polyadenylation redundantly with flowering time control protein (FCA), another RBP [99]. FPA and FCA were further shown to be involved in transcription termination limiting intergenic transcription for a wide array of genes [100]. FPA controls cleavage and polyadenylation of ETHYLENE RESPONSE FACTOR 4 (ERF4) during exposure to the bacterial flagellin peptide elicitor flg22 to limit the defense response to bacterial infection [101].

### 3.2. Trafficking and Translocation

RBPs ensure that mature RNAs are transported to the correct cellular locale. In rice endosperm, it was shown that two zipcode RBPs binding glutelin mRNA form a quaternary structure with two membrane fusion factors [102]. RBP-P and RBP-L, as discussed in Section 2.1.1, participate in the coopting of endosomal trafficking for mRNA translocation [103,104]. Mutant lines of RBP-P proteins in rice showed mis-localized glutelin and prolamine mRNAs (coding for the two most prominent seed storage proteins), as well as altered regulation of biological processes during seed development [104].

The nucleoporin complex participates in the proper apportioning of mRNAs between the nucleus and cytoplasm in response to stresses, mediated by the RBPs MOS2, MOS3, and MOS11. These MOS factors were originally identified as modifiers of snc1 (suppressor of NPR1-1, constitutive1), which encodes a resistance gene involved in basal plant immunity [105]. MOS2 contains a G-patch motif and Kyprides, Ouzounis, Woese (KOW) domains, indicating that it binds RNA. Mutant lines showed suppressed constitutive defense responses, and MOS2 has been predicted to play a role in RNA processing [106,107]. MOS3 is localized to the nuclear envelope, where it is involved in nuclear mRNA export with a role in innate immunity [106]. MOS11 has homology to a human RNA binding protein (cytokine-inducible protein 29, or CIP29) and is localized to the nucleus, where it acts in mRNA nuclear export [108]. A plant homolog of a component of the transcription export complex in yeast and humans, heparanase 1 or HPR1, is further involved in mRNA export and defense signaling [109]. The SARs (suppressor of auxin resistance) are localized to the nuclear membrane, acting as nucleoporins and mediating export of polyadenylated RNA [110].

In angiosperms, certain mRNAs involved in organ development are translocated through the phloem as long-distance signaling agents in the form of ribonucleoprotein complexes mediated by RBPs [111]. CmRBP50, a polypyrimidine tract binding protein, is essential for the assembly of one such complex based on the phosphorylation of phosphoserine residues at the C terminus [112]. Three proteins bind directly with phosphorylated CbRBP50 to assemble a complex that binds mRNA containing polypyrimidine tract binding motifs for transport through the phloem sieve tube system [112]. In pumpkin, another polypyrimidine tract binding protein, RBP50, forms ribonucleoprotein complexes with phloem-mobile mRNAs [113].

### 3.3. Chaperoning

RNA chaperones play a significant role in cold acclimation by binding RNAs nonspecifically and modulating their secondary structure during chilling conditions [114]. These proteins are typically from the glycine-rich RNA-binding protein (GRP) family, a group of developmentally regulated RBPs that possess N-terminal RNA recognition motifs (RRMs) and often exhibit induction by low temperatures. A family of such genes was identified in plant mitochondria via affinity chromatography, diverging in their C-terminal sequence; this indicates that while they may recognize RNA through the same mechanism, they target diverse areas of RNA biology for regulation [11]. One of these mitochondrially located proteins, GR-RBP3, was found in cucumber to be highly expressed during chilling and to minimize the effects of stress such as slowed growth speed, electrolyte leakage, and reactive oxygen species [115]. Overexpression of GR-RBP3 also upregulated nine Arabidopsis genes involved in stress defense; together, these results imply that GR-RBP3 plays a role in maintaining mitochondrial function during low temperature and thereby increasing cold stress resistance [115]. Another GRP, termed AtRZ-1a, showed remarkably similar patterns of expression to GR-RBP3 under stress, and knockout and overexpression lines showed that atRZ-1a plays a key role in cold stress defense [116]. The GRPs are heavily regulated by stress conditions, including cold, salt, and dehydration [117].

In cotton, between 32 and 37 GRPs have been identified and subdivided into four families based on the arrangement of glycine repeats and the presence of other motifs [118]. Gene expression analysis in cotton and Chinese cabbage (*Brassica rapa*) showed that these GRPs participate in response to various abiotic stresses in different tissues according to developmental stage [118,119]. A member of this protein family, GRP8, has also been shown to participate in root hair cell determination in a phosphate starvation-dependent manner by binding to and promoting the abundance of WRKY DNA-BINDING PROTEIN 75 (WRKY75) [57]. GRP8 binds to a TG-rich motif and promotes the abundance of the mRNAs of phosphate transporters.

In *Arabidopsis thaliana*, the universal stress protein UDP-Sugar pyrophosphorylase (AtUSP) acts as an RNA chaperone to bind nucleic acids nonspecifically, showing high nucleic acid-melting activity, to help maintain the RNA secondary structure during cold temperatures and maintain gene expression [120].

### 3.4. Stability and Decay

Bruno-RBPs AtBRN1 and AtBRN2 in Arabidopsis bind the 3’ UTR of the flowering pathway integrator SUPPRESSOR OF OVEREXPRESSION OF CONSTANS1 (SOC1), which is regulated by FLC, to repress SOC1 via mRNA decay pathways to control flowering time [13,121]. Overexpression lines of these Bruno-RBPs showed delayed flowering time even when crossed with SOC1 overexpression lines provided the 3’ UTR was included, whereas an AtBRN1 AtBRN2 double mutant line showed early flowering [121].

In chloroplasts and mitochondria, transcripts are protected against degradation by exonucleases by RBPs at their 5’- and 3’-termini [122,123]. Such protection leaves behind a small RNA footprint after RNA degradation, which has been used to probe the organellar RBP binding using the sRNA miner software and reveal differential protective patterns in mitochondria compared to chloroplasts [124]. Mitochondrial transcripts showed a bias for protection at the 3’ end, whereas chloroplast transcripts showed no such bias [124]. The nuclear-encoded chloroplast-targeted RBP pentatricopeptide repeat-containing protein 10 (PPR10) protects two intergenic RNA regions overlapping by 25 nucleotides to protect the RNA against degradation [122].

The nuclear-encoded protein termed S1 domain containing RBP (SDP), which is chloroplast-targeted, binds the chloroplast ribosomal RNAs 23S, 16S, 5S, and 4.5S and possesses RNA chaperone activity, indicating that SDP may play a role in ribosomal RNA processing in chloroplasts [125]. SDP knockout mutants show decreased survival rate during salt, heat, UV, and cold stress as well as altered expression of nuclear stress-responsive genes, indicating that SDP plays a role in abiotic stress response [126]. These SDP mutants also showed slowed germination, dwarfism, chlorophyll a deficiency, and abnormal chloroplast structures [125].

The protein SERRATE (SE) was identified by PIP-seq and RNA-chromatography as an inhibitor of root hair cell determination via miRNA biogenesis [57]. SE binds to a GGN repeat motif, and independently of its inhibitory role promotes the stability of several mRNAs involved in root hair cell length [57]. The RRM-containing protein AtRBP45b may play a role in RNA metabolism, specifically RNA stability, through interaction with cap-binding protein CBP20 and polyA binding protein PAB8 [127]. AtRBP45b is found in the nucleus and cytoplasm and has auxiliary domains related to protein–protein interaction, likely mediating its interaction with CBP20 and PAB8 [127].

In rice, 3′-UTR-interacting protein 1 (UIP1) was identified as an RBP through yeast three-hybrid screening [128]. UIP1 interacts with the 3’ UTR of the RuBisCo small subunit (rbcS) mRNA to mediate its stress-induced mRNA decay under drought and salt stress conditions. Rice overexpression lines showed increased resistance to salt, drought, and chilling stress [128].

### 3.5. Gene Silencing

RBPs partner with small RNAs to silence retrotransposons and repetitive DNA elements through the formation of heterochromatin [129]. This silencing involves AGO1, AGO10 (Argonaute proteins), katanin (microtubule severing enzyme), and VCS (decapping component) [129]. Small RNAs involved in this process are stabilized in plants by 3’ termini methylation, protecting them uridylation-targeted degradation mediated by the nucleotidyl transferases HESO1 and HEN1 [130,131].

### 3.6. Viral RNA Suppression

Viral genomes rely on many RBPs of host origin for their replication and dissemination [49]. Consequently, host RBPs play a role in defense against viral pathogens; the Arabidopsis pumilio RNA binding protein 5 (APUM5) provides protection against cucumber mosaic virus (CMV) by directly binding to the 3’ UTR of CMV RNAs to repress CMV replication [4,132]. Another RBP, binding to ToMV RNA 1 (BTR1), binds RNA around the initiation codons of the replication genes for tobacco mosaic virus (ToMV) to inhibit ToMV replication, as demonstrated by BTR1 knockout and overexpression lines [133]. Antiviral RNA silencing in plants occurs via the production of small RNAs from viral RNAs by dicer-like protein 4 (DCL4), as targeted by the interacting partner of DCL4, double-stranded RNA-binding protein 4 (DRB4), which binds double-stranded RNA of turnip yellow mosaic virus (TYMV) [134]. DRB4 is induced during viral infection and exported from the nucleus to the cytoplasm, where it associates not only with viral RNA but with a viral translational enhancer to repress viral protein accumulation [134]. In *Capsicum annuum*, pathogenesis-related protein 10 (CaPR-10) functions as a ribonuclease targeting tobacco mosaic virus (TMV) RNA; CaPR-10 is induced upon TMV infection, after which it is phosphorylated to increase its ribonucleolytic activity and increase its cleavage of viral RNAs [135]. Thus, RBPs are critically involved in multiple mechanisms of plant defense against viral infection: translational repression, ribonucleolytic cleavage, and RNA interference.

### 3.7. Other Plant RBPs

Other plant RBPs or putative RBPs have been identified, from a variety of species, whose impact on the life of their RNA targets has not yet been elucidated. Those discussed briefly hereafter demonstrate the importance of RBPs in regulation of stress response and development, particularly in germination and flowering.

In Arabidopsis, the ABA-regulated RNA-binding protein ARP1 was identified as an ABA-responsive protein localized to the nucleus [136]. ARP1 is downregulated by ABA, seems to modulate the transcript levels of several genes involved in gene regulation, and both overexpression and knockout lines showed delayed germination during ABA, salt, and dehydration conditions, indicating that ARP1 plays a role in posttranscriptional RNA regulation during germination [136]. Similarly, the mei2 C-terminal RRM only protein MCT1 is localized to the nucleus to impact the expression of several ABA-related genes [137]. Overexpression and knockout lines for this protein demonstrated that, conversely to ARP1, it is upregulated under ABA treatment and inhibits germination and greening, suggesting that it plays an inhibitory role in germination and seedling growth, likely mediated by its RNA binding domain. However, no such binding activity has been directly observed as yet [137].

Another putative RBP involved in seed germination was identified in *Malus prunifolia*. The glycine-rich protein MpGR-RBP1 was predicted as an RBP that is upregulated during salt, oxidative, or ABA stress [138]. An Arabidopsis overexpression line demonstrated that MpGR-RBP1 is involved in seed germination; this line showed accelerated germination under salt and oxidative stress, and enhanced salt tolerance as measured by electrolyte leakage, chlorophyll concentration, ROS accumulation, and stomatal closure [138]. Similarly, the *Aeluropus littoralis* Stress-Related Gene 1 (AlSRG1) gene encodes a protein involved in abiotic stress response that contains an RRM motif [139]. In tobacco overexpression lines, AlSRG1 improved resistance to several abiotic stresses and reproduced successfully under conditions that killed the control plants before flowering, and showed higher levels of the transcripts of genes related to ROS-scavenging and stress-related transcription factors, indicating that AlSRG1 plays a positive role in abiotic stress response [139].

A putative RBP isolated from hot pepper (*Capsicum annuum* cv. *Bukwang*) termed CaRBP is also localized to the nucleus—specifically to the nucleolus—and functions in transgenic tomato to delay flowering and alter the expression of various genes related to flowering [140]. Given its nucleolar localization and predicted RNA binding capacity, CaRBP may be involved in ribosome biogenesis or RNA metabolism.

These putative RBPs represent a rich potential for the characterization of the roles of RBPs in RNA biogenesis, processing, and degradation in plants. Techniques like those discussed in Section 2 are key for tying these RBPs and others like them to the processes they regulate in their target RNAs.

## 4. Manipulation of RBPs Confers Desirable Traits in Plants

Due to their widespread nature and the role they play in abiotic stress response, RBPs and their RNA partners are key targets for biotechnological development in plants. Furthermore, understanding RNA–protein regulatory networks will facilitate successful application of plant biotechnology in general, because of the significant genotype to phenotype barrier that can be presented by highly redundant and interlinked endogenous networks [141]. All genes and their mRNAs are part of these networks, regulated at every level by RBPs. A deeper understanding of those networks will allow judicious selection of the smallest number of key intervention points necessary to produce desired phenotypes.

Several RBPs have already been identified as important biotechnological targets in various plant species. The glycine-rich proteins, in particular, seem of great interest; AtGRP2 and AtGRP7 were expressed in rice, showing similar phenotype during salt or cold stress, but faster recovery and higher grain yields compared to controls during drought stress [142]. GRP8 is a target for biotechnological advancement in engineering stress-resistant plants due to its role in upregulating phosphate uptake and biomass accumulation and the fact that overexpression lines do not exhibit negative aerial phenotypes [57]. The GRP MhGR-RBP1 was identified as a putative RBP in *Malus hupehensis*, where its transcript levels are highly regulated by various abiotic stresses, indicating that it may be involved in abiotic stress response and a potential target for biotechnological development [143].

RNA chaperones are also targets of interest. A bacterial cold shock protein expressed in plants conferred improved growth in Arabidopsis and improved grain yield under drought stress in Maize [144]. Field trials of maize expressing this heterologous cold shock protein showed a 6% yield increase across trials due to increased drought resistance [145].

In sugar beet, six genes coding for RBPs were identified as being able to increase salt tolerance in yeast [146]. Two of these genes participate in splicing, and the other four have only been putatively assigned as being involved in RNA metabolism. One of these salt-tolerance genes, BvSATO1 (which is involved in splicing), was verified in sugar beet and Arabidopsis, which showed that BvSATO1 increased salt tolerance in Arabidopsis [146].

In Arabidopsis, the RBP AtRGGA is involved in the proper response to osmotic stress [147]. Overexpression of AtRGGA conferred increased resistance to ABA and salt stress in Arabidopsis due to modification of the transcriptome [147]. In apple, overexpression of the RBP MhYTP1 improved drought resistance as measured by photosynthesis and water use efficiency [148]. Further discovery of such RBPs will play an important role in biotechnological advancement in plants.

## 5. Conclusions and Perspectives

The recent application of RIC in plants has provided a holistic view of the repertoire of poly(A)-mRNA interacting RBPs in plant cells. The results of these studies have recently been reviewed [1], so they will not be discussed in detail here. However, we will note that these studies vastly expanded the number of proteins we can confidently assign as RBPs or candidate RBPs to include many that do not contain a canonical RNA binding domain. Together, these initial studies identified 2701 unique RNA-bound proteins (836 of which are classified as RBPs and 1865 of which are classified as putative RBPs), making up ~6.7% of the Arabidopsis proteome, which was recently estimated at ~18,000 unique proteins [1,149]. Despite their relative commonness, only 18 RBPs were identified by all studies, highlighting the responsiveness of the RBPome to different growth conditions and stresses and suggesting that we have only uncovered a portion of the proteins that may bind RNA under specific circumstances.

Taken together with the studies reviewed above, an image emerges of the intimate relationship between RBPs and RNA, in which RNAs are accompanied by RBPs during every stage of their existence—from the initiation of transcription to their eventual degradation. RBPs have been observed to play important roles in such diverse physiological processes as plant development, immunity, abiotic stress response, and silencing of repetitive DNA elements by interacting with every stage of RNA existence. Indeed, given their ubiquitous and widespread nature in RNA biology, it seems more reasonable to expect that RNA–protein interactions play key roles in the regulation of every biological process. These studies hint at a highly complex regulatory network of ribonucleoprotein complexes whose memberships and activities are constantly in flux in response to developmental stage and external stimuli, each factor regulating and being regulated multiply depending on the status of the network. The conception of a ‘naked’ RNA, sans any RBP companions, may be entirely mythic.

What does this mean for the study of plant molecular biology? In short: RNA and protein interactions are everywhere, and they should be expected to be everywhere. For any given gene of interest, probing the RNA interactome of the protein and the protein interactome of the RNA is likely to yield interesting information regarding the gene’s regulation, and both should be considered as key components of routine study. This is currently hindered by the fact that there are no RNA-to-protein methods for individual RNAs that have been used in plants—the application of such methods developed in other systems, such as ChIRP-/RAP-MS or TRAP-MS, should be prioritized, as the current bias toward protein-to-RNA methods likely introduces certain myopias in our knowledge. Moreover, the RNA–protein interactions of most factors should be expected to change in response to developmental stage or environmental stimuli, such as biotic or abiotic stress. These are not static relationships.

As nodes of this regulatory network are further illuminated, it will be interesting to discover how the individual regulatory systems currently understood are interlinked with each other to broadly govern plant physiology. The observation of many metabolic enzymes moonlighting as RBPs in both plants and animals likely represents another category of such interlinks, tying metabolic regulatory systems to stress response regulation. The exact functions of these metabolic RBPs, if any, remain to be discovered, but show promise to fulfill the predictions of the REM hypothesis.

Beyond the moonlighting of metabolic enzymes themselves, the prevalence of RBPs or candidate RBPs that lack canonical RNA binding domains in the interactome capture studies suggests that we may see similar ‘moonlighting’ activities emerge from other categories of proteins, and that RNA/protein interactome studies might be warranted even in cases where no known connection to RNA processing exists. By all evidence, RNA–protein interactions seem ubiquitous and critical for fine-tuned control of development and environmental response.

## Figures and Tables

**Table 1:** Summary of RNA-Protein Methods and their use in Plants.

Method	Pros	Cons	Plant Refs
*Protein-to-RNA*			
RIP-seq	No genetic trans., reversible crosslinking, well established in plants, no radiolabeling	Uses antibody–antigen interaction, non-specific crosslinking, large amounts of starting material, no info on RBP site	He et al., 2009, Streitner et al., 2012; Yin et al., 2012; Rowley et al., 2013; Bardou et al., 2014; Francisco-Mangilet et al., 2015; Xing et al., 2015; Bazin et al., 2018; Marmisolle et al., 2018; Schmid et al., 2019; Tian et al., 2019
CLIP-seq	No genetic trans., provides info. on the RBP binding site, well-established in plants	Uses antibody–antigen interaction, uses radiolabeling, large amounts of starting material	Meyer et al., 2017; Zhang et al., 2015
HITS-CLIP	Increased coverage	*As CLIP-seq*	Zhang et al., 2015
PAR-CLIP	More efficient UV-crosslinking	*As CLIP-seq,* favors certain RBP-RNA interactions	*None*
iCLIP	Increases precision of RBP site prediction	*As CLIP-seq*	Meyer et al., 2017
dCLIP	Permits comparisons across all CLIP exps.	*As CLIP-seq*	*None*
uvCLAP	Tight binding affinity, uniform pulldown efficiency, quantify background, no radiolabeling, no antibodies	Not in plants, needs genetic trans., may alter RNA–protein interactions, no info on RBP site, large amounts of starting material	*None*
TRIBE/HyperTRIBE	No pull down, small amounts of starting material, no radiolabeling, no antibodies	Not in plants, needs genetic trans., editing occurs in a wide range around the binding site, no info on RBP site	*None*
*RNA-to-Protein*			
ChIRP-MS/RAP-MS	High affinity interaction, no genetic trans., no radiolabeling, no antibodies	Not in plants, no info on RBP site, large amounts of starting material	*None*
RNA Small Molecule Labeling	No genetic trans., no radiolabeling, no antibodies	In vitro only	*None*
RNA Nucleotide Substition	No genetic trans., no radiolabeling, no antibodies	In vitro only	*None*
RNA Aptamer Pulldown	High affinity interaction, many aptamers, no radiolabeling, no antibodies	Not in plants, needs genetic trans., no info on RBP site, may alter RNA–protein interactions, large amounts of starting material, may be prone to aggregation	*None*

**Table 2:** Summary of Plant RBPs Identified by Baited RNA-Protein Approaches.

RBP	Plant System	Method	Number of RNA Targets	References
AGO4	*Arabidopsis thaliana*	RIP	2	Wierzbicki et al., 2009
AtGRP7	*Arabidopsis thaliana*	RIP-seq/iCLIP	452/858	Streitner et al., 2012; Meyer et al., 2017
AtNSRa	*Arabidopsis thaliana*	RIP-seq	>2000	Bardou et al., 2014; Bazin et al., 2018
AtNSRb	*Arabidopsis thaliana*	RIP-seq	>2000	Bardou et al., 2014; Bazin et al., 2018
CPsV 24K *(viral)*	*Nicotiana benthamiana*	RIP	2	Marmisolle et al., 2018
CPsV 24K *(viral)*	*Nicotiana benthamiana*	RIP	2	Marmisolle et al., 2018
CSP1	*Arabidopsis thaliana*	RIP-chip	>6000	Juntawong et al., 2013
IDN2	*Arabidopsis thaliana*	RIP	1	Zhu et al., 2013
FCA	*Arabidopsis thaliana*	RIP	1	Tian et al., 2019
HLP1	*Arabidopsis thaliana*	HITS-CLIP	>5000	Zhang et al., 2015
KTF1	*Arabidopsis thaliana*	RIP	1	He et al., 2009
NSF	*Oryza sativa*	RIP	?	Tian et al., 2020
PUMPKIN	*Arabidopsis thaliana*	RIP-seq	5	Schmid et al., 2019
PDM1	*Arabidopsis thaliana*	RIP	1	Yin et al., 2012
Rab5a	*Oryza sativa*	RIP	?	Tian et al., 2020
RBP-L	*Oryza sativa*	RIP	?	Tian et al., 2020
RBP-P	*Oryza sativa*	RIP	?	Tian et al., 2020
SR45	*Arabidopsis thaliana*	RIP-seq	>4000/>1800	Xing et al., 2015; Zhang et al., 2017
THO2	*Arabidopsis thaliana*	RIP	6	Francisco-Mangilet et al., 2015

## Data Availability

No new data were created or analyzed in this study. Data sharing is not applicable to this article.

## Supplementary Materials

The following are available online at https://www.mdpi.com/1422-0067/22/6/2845/s1, Table S1: Summary of Plant RBPs Identified by Classical Methods.
