# Supplementary material for: The Landscape of RNA-Protein Interactions in Plants: Approaches and Current Status
Source: Int J Mol Sci. 2021 Mar 11;22(6):2845. doi: 10.3390/ijms22062845 (PMC7999938; doi:10.3390/ijms22062845)
Supplement: Supplementary file 1 [file ijms-22-02845-s001.pdf]

Supplementary Table 1. Summary of Plant RBPs Identified by Classical Methods

RBP	Role	References
AlSRG1	*Abiotic stress response*	Ben Saad et al., 2018
APUM5	*Repress viral replication*	Huh and Paek, 2013b
ARP1	*Modulation of transcript levels*	Jung et al., 2013
AtBRN1	*Control flowering time*	Kim et al., 2013
AtBRN2	*Control flowering time*	Kim et al., 2013
AtGRP7	*Modulation of alternative splicing*	Schoning et al., 2007
AtGRP8	*Modulation of alternative splicing*	Steffen et al., 2019
AtRBP45b	*RNA stability*	Muthuramalingam et al., 2016
AtRZ-1a	*Cold stress defense*	Kim et al., 2005
AtUSP	*RNA chaperoning*	Melencion et al., 2017
BTR1	*Repress viral replication*	Fujisaki et al., 2008
CaPR-10	*Cleavage of viral RNAs*	Park et al., 2004
CaRBP	*Delay flowering*	Kim et al., 2016
CmRBP50	*Long distance RNA translocation*	Li et al., 2011
Dek42	*Modulation of alternative splicing*	Zuo et al., 2019
DRB4	*Repress viral protein accumulation*	Jakubiec et al., 2012
FCA	*Alternative polyadenylation, transcription termination*	Hornyik et al., 2010; Sonmez et al., 2011
FPA	*Alternative polyadenylation, release of flowering repression, limit defense response*	Hornyik et al., 2010; Lyons et al., 2013
GR-RBP3	*RNA chaperoning, cold resistance*	Wang et al., 2018
GRP8	*Root hair cell determination*	Foley et al., 2017
HEN1	*RNA stability*	Ren et al., 2012
HESO1	*RNA stability*	Zhao et al., 2012
HPR1	*mRNA nuclear export, defense signaling*	Pan et al., 2012
MCT1	*Modulation of gene expression*	Gu et al., 2016
MOS2	*Nucleoporin trafficking of RNAs, plant innate immunity*	Zhang et al., 2005; Monaghan et al., 2010
MOS3	*Nucleoporin trafficking of RNAs*	Zhang et al., 2005; Monaghan et al., 2010
MOS11	*Nucleoporin trafficking of RNAs*	Germain et al., 2010; Monaghan et al., 2010
MOS12	*Splicing of resistance genes*	Xu et al., 2012
MpGR-RBP1	*Germination, salt stress resistance*	Tan et al., 2014
MtNSR1	*Modulation of splicing*	Campalans et al., 2004
PPR10	*RNA stability*	Pfalz et al., 2009
RBP50	*Long distance RNA translocation*	Ham et al., 2009
RBP-L	*Subcellular trafficking of mRNAs*	Tian et al., 2018; Tian et al., 2020
RBP-P	*Subcellular trafficking of mRNAs*	Tian et al., 2018; Tian et al., 2020

SARs	*Nucleoporin trafficking of RNAs*	Parry et al., 2006
SDP	*RNA chaperoning, stress resistance*	Han et al., 2015
SERRATE (SE)	*Inhibition of root hair cell determination, RNA stability*	Foley et al., 2017
UIP1	*Mediation of RNA decay, abiotic stress resistance*	Park et al., 2013
